# Supplementary material for: Content-rich biological network constructed by mining PubMed abstracts
Source: BMC Bioinformatics. 2004 Oct 8;5:147. doi: 10.1186/1471-2105-5-147 (PMC528731; doi:10.1186/1471-2105-5-147)
Supplement: Additional File 2 — The original results of the above study (non-essential files are deleted to keep the file size under the limit set by BMC bioinformatics). [file 1471-2105-5-147-S2.bz2 › chilibotAdditionalFile2/dip10/597ID8524402E10973/html/PPP3CA_PPP3R1.html]

 


 **PPP3CA** and **PPP3R1** 
  
Found 30 abstracts in PubMed, retrieved 10.  
 

 What does Google say? 
 PDF only 
| .edu only 

---

**Interactive relationship** (e.g. stimulation, inhibition, etc)

**Inhibitory relationship**- The Ca2 dependent binding of phage displayed peptides to CnB  [ **PPP3R1** ]  and CaM is specifically blocked by synthetic peptides derived from the CnB  [ **PPP3R1** ]  binding domain of CnA  [ **PPP3CA** ]  and the CaM binding domain of myosin light chain kinase respectively.  Ref: 10072758 Gene, 1999
- The deduced amino acid sequence well conserves the calcineurin B CnB  [ **PPP3R1** ]  binding domain and two subdomains, a calmodulin binding and an autoinhibitory domain, showing 77 85% and 82% identities to the isoforms of Drosophila melanogaster CnA  [ **PPP3CA** ]  and human CnA  [ **PPP3CA** ] , respectively.  Ref: 11886782 Insect Biochem Mol Biol, 2002
**Neutral relationship**- Its crystal structure reveals that the C terminal of CNB  [ **PPP3R1** ]  lies in close vicinity of the N terminal of CNA  [ **PPP3CA** ]  and each end has a long arm not involved in the active site.  Ref: 12890484 Biochem Biophys Res Commun, 2003
- In kappa CnG, 148 and 168 codons coincide with the corresponding codons of kappa CnB  [ **PPP3R1** ] , and 136 and 167 codons correspond to kappa CnA  [ **PPP3CA** ] .  Ref: 9119217 Genetika, 1996
- A yeast two hybrid system has demonstrated the molecular interaction between B. mori CnA  [ **PPP3CA** ]  and CnB  [ **PPP3R1** ] .  Ref: 11886782 Insect Biochem Mol Biol, 2002

**Non-interactive relationship** (e.g. studied together, co-existance, homology, etc.)

- Calcineurin CN is a Ca 2 calmodulin CaM dependent serine threonine protein phosphatase which is a heterodimer composed of a 61kDa catalytic subunit CNA  [ **PPP3CA** ]  and a 19kDa regulatory subunit CNB  [ **PPP3R1** ] .  Ref: 12890484 Biochem Biophys Res Commun, 2003
- The binding preferences identified with peptide libraries are consistent with the features of the CnB  [ **PPP3R1** ]  binding domains of all CnA  [ **PPP3CA** ]  isoforms and the CaM binding domains of CaM targets.  Ref: 10072758 Gene, 1999
- Here we report the identification and characterization of calcineurin genes in Caenorhabditis elegans cna 1  [ **PPP3CA** ]  and cnb 1  [ **PPP3R1** ] , which share high homology with Drosophila and mammalian calcineurin genes.  Ref: 12221132 Mol Biol Cell, 2002
- The results of Southern blotting experiments are compatible with the existence of multiple genes for CNA  [ **PPP3CA** ]  and a single gene for CNB  [ **PPP3R1** ] .  Ref: 11071287 Mol Biochem Parasitol, 2000
- The use of several restriction endonucleases allowed three alleles of kappa casein kappa CnA  [ **PPP3CA** ] , kappa CnB  [ **PPP3R1** ] , kappa CnF to be typed and new allele variants in yak, European bison, and buffalo to be revealed.  Ref: 9119217 Genetika, 1996
- This suppression was found to be due to inhibition of interactions between activated calcineurin Cn and nuclear factor of activated T cells NF AT, inhibition of cleavage of the autoinhibitory domain of the CnA  [ **PPP3CA** ]  subunit, and inhibition of heterodimer formation by CnA  [ **PPP3CA** ]  and CnB  [ **PPP3R1** ]  subunits, resulting in the absence of NF AT in nuclei of the T cells.  Ref: 9182928 Arthritis Rheum, 1997
- The protein is a heterodimer of one catalytic CnA  [ **PPP3CA** ]  subunit and one regulatory CnB  [ **PPP3R1** ]  subunit.  Ref: 9199180 Biochem Biophys Res Commun, 1997
- Northern blot analyses revealed that both CnA  [ **PPP3CA** ]  and CnB  [ **PPP3R1** ]  genes were expressed in various larval and adult tissues of B. mori.  Ref: 11886782 Insect Biochem Mol Biol, 2002
